# Computational analysis of fused co-expression networks for the identification of candidate cancer gene biomarkers

**DOI:** 10.1038/s41540-021-00175-9

**Published:** 2021-03-12

**Authors:** Sara Pidò, Gaia Ceddia, Marco Masseroli

**Affiliations:** Department of Electronics, Information and Bioengineering, Politecnico di Milano, Milan, Italy

**Keywords:** Software, Cancer

## Abstract

The complexity of cancer has always been a huge issue in understanding the source of this disease. However, by appreciating its complexity, we can shed some light on crucial gene associations across and in specific cancer types. In this study, we develop a general framework to infer relevant gene biomarkers and their gene-to-gene associations using multiple gene co-expression networks for each cancer type. Specifically, we infer computationally and biologically interesting communities of genes from kidney renal clear cell carcinoma, liver hepatocellular carcinoma, and prostate adenocarcinoma data sets of The Cancer Genome Atlas (TCGA) database. The gene communities are extracted through a data-driven pipeline and then evaluated through both functional analyses and literature findings. Furthermore, we provide a computational validation of their relevance for each cancer type by comparing the performance of normal/cancer classification for our identified gene sets and other gene signatures, including the typically-used differentially expressed genes. The hallmark of this study is its approach based on gene co-expression networks from different similarity measures: using a combination of multiple gene networks and then fusing normal and cancer networks for each cancer type, we can have better insights on the overall structure of the cancer-type-specific network.

## Introduction

Cancer is a complex disease affecting various biological processes in human cells and causing abnormal cell growth, invasion, and migration^1^. Since human cells are complex biological systems, changes occurring for cancer disease may appear at different levels of the cell organization^1^. Thus, system biology is one of the most commonly used approaches for understanding such complexity since it studies the molecular interactions determining particular biological functions within a cell^1–5^. Biological networks are the typical computational models used in system biology for understanding the functional mechanisms in a cell as they can provide insights about the overall structure of the molecular interactions^1–3^. Gene co-expression networks are the most commonly studied biological networks in network inference (Supplementary Material Section Gene co-expression network inference), as their application is suitable for large data sets and their construction is condition-specific, i.e., they derive from case-specific gene expression data^6^. Gene co-expression network analysis has been applied in various biological studies, especially in cancer-based research in order to identify candidate cancer biomarkers, i.e., significant molecules related to cancer development and their interactions^4,5,7–9^. Gene expression studies have shown that diverse gene expressions are associated with fundamental differences in clinical and biological features^10,11^. The majority of these studies focused on the identification of individual cancer biomarkers and their prognostic use, without addressing the main objective of system biology, i.e., a better comprehension of the functional mechanisms of the found biomarkers^6,12–16^. Gene co-expression networks are the best way to address this aim, leveraging on both biological networks and gene expression studies.

Here, we propose a novel general approach based on the construction of multiple gene co-expression networks and its implementation in a computational framework to identify interesting gene biomarkers and consequently better distinguish clinical outcomes across cancer types. Using data from The Cancer Genome Atlas (TCGA)^17^, we demonstrate the relevance of the approach by applying it to three representative cancer types. The novel hallmark of our method is the integration of multiple gene co-expression networks for each cancer type. Indeed, a single type of co-expression network is commonly used in the literature^18^; conversely, in this work, we use and combine two different measures for the computation of co-expression values. This allows inferring diverse features, depending on the chosen similarity measures that derive from the expression profiles. We compare our results with those from both a state-of-the-art method for finding gene biomarkers and a single type of gene co-expression network. We also perform knowledge-based systematic evaluations on the extracted gene biomarkers to evaluate their involvement in the cancer-type progress. Particularly, we assess their relevance from the perspective of the gene set enrichment analysis, the literature and their drug actionability. Our results reveal that the integration of multiple gene co-expression networks leads to the identification of new promising prognostic gene modules; this demonstrates that our new approach contributes to the comprehension of the molecular mechanisms related to candidate gene biomarkers by using a novel perspective on gene co-expression networks. We also compare our results with the ones from differential gene expression analysis, which is the usual baseline method to identify biomarkers in biology^19^.

## Results

In this section, we present the results obtained with our approach applied on TCGA data of KIRC, LIHC, and PRAD cancer types. We report the communities of genes identified in the fused network for each cancer type and the evaluation of their relevance, both computationally and biologically.

### KIRC, LIHC, and PRAD networks

We apply our computational approach on the TCGA gene expression data for KIRC, LIHC, and PRAD cancer types to build their gene co-expression networks. Table 1 illustrates the number of genes in the original TCGA data and after our pre-processing step. On average, 20% of RNA (protein-coding or long non-coding) genes and 81% of miRNAs are extracted. These genes are the nodes of all the networks built in the main steps of our developed pipeline, whose number of edges is reported in Table 2. In particular, edges in the Euclidean distance networks for the normal and cancer condition are those after their 99th percentile thresholding, whereas edges in the Pearson’s correlation networks are those filtered using the permutation method and a p-value percentile threshold such that their number is fairly homogeneous across the similarity measures. Notably, the number of edges in the union of the two similarity networks for each condition (Normal and Cancer) is very similar (less than 1% difference on average overall) to the sum of the edges in the Euclidean distance and Pearson’s correlation networks for each condition (respectively fifth and sixth vs. seventh and eighth row of Table 2); thus, almost all gene relations identified with the two similarity measures regard different gene pairs. Conversely, the sum of the edges of the two Normal and Cancer networks is much greater than the number of edges in their union (Merged) network (KIRC 129%, LIHC 130%, PRAD 128%), with Normal networks that have more edges than the Cancer ones (about 27% more on average). The relevant edges selected with the disparity filter in the final (Fused) networks are on average about 4.9% of the edges in the Merged ones.

**Table 1:** Number of RNA genes and miRNAs.

Genes	KIRC	LIHC	PRAD
Original RNA	60,483	60,483	60,483
Original miRNA	1747	1747	1747
Filtered RNA	12,792	11,595	12,097
Filtered miRNA	1397	1421	1411
Filtered Total	14,189	13,016	13,508

RNAs and miRNAs in the initial samples (Original) and in the final ones (Filtered) used for network construction.

**Table 2:** Number of edges in each network.

Network	KIRC	LIHC	PRAD
Normal Euclidean	1,006,639	847,082	912,331
Cancer Euclidean	1,006,639	847,082	912,331
Normal Pearson	1,979,654	1,738,560	1,743,387
Cancer Pearson	1,124,575	1,218,642	1,298,722
Normal	2,971,772	2,530,341	2,622,268
Cancer	2,129,102	2,042,152	2,209,407
Normal Euclidean+Pearson	2,986,293	2,585,642	2,655,718
Cancer Euclidean+Pearson	2,131,214	2,065,724	2,211,053
Merged	3,954,994	3,517,068	3,770,395
Normal+Cancer	5,100,874	4,572,493	4,831,675
Fused	201,314	169,404	182,453

From the single measure (Euclidean and Pearson) networks, through the union of the two measures (Normal and Cancer) networks, until the union of the normal and cancer networks (Merged) and their fusion (Fused).

### Topological evaluation of fused networks

We evaluate each fused network by using the topological measure average degree, which helps to understand the network architecture; it is the average number of edges that each node has in the network. The average degree of the KIRC, LIHC, and PRAD fused networks is 28.38, 26.03, and 27.01, respectively, while their number of nodes and edges is reported in the last row of Tables 1 and 2, respectively. As expected, since we prune the networks using the disparity filter (Network Fusion panel in Fig. 5), the average degree is very low compared to the number of nodes. Moreover, it is very similar across cancer types, confirming the homogeneity given by our data processing.

**Fig. 1:**
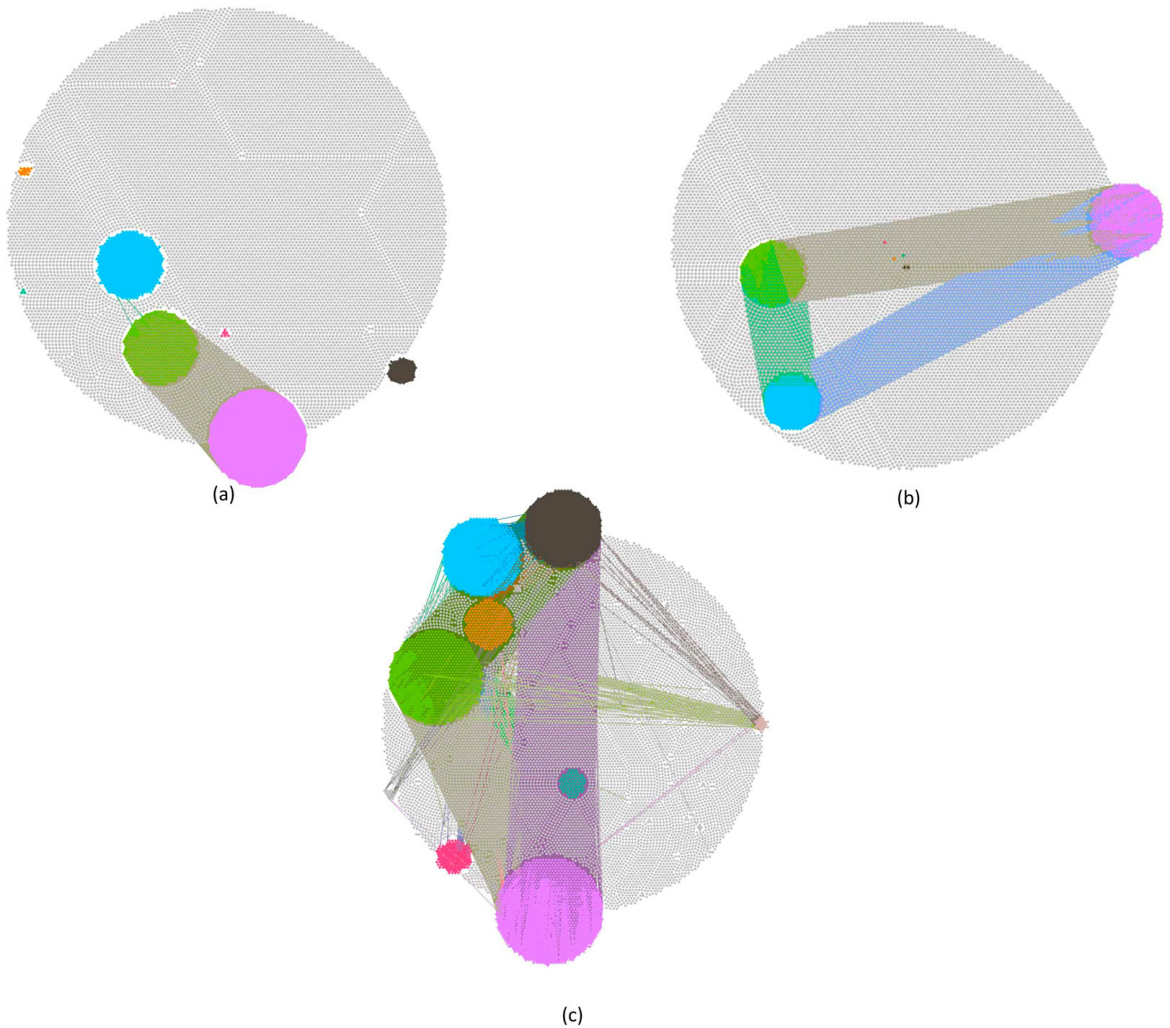
Gene co-expression networks. **a** KIRC, **b** LIHC, and **c** PRAD gene co-expression networks regarding fused normal and cancer data. Different colors represent different communities of genes and their relations; these communities are found using the modularity method implemented in the Gephi software.

In the KIRC, LIHC, and PRAD fused networks we identify 6, 3, and 9 gene communities, respectively; Fig. 1 shows them in different colors. In these networks, overall they include 1,543 KIRC, 839 LIHC, and 3502 PRAD genes (the network IC genes), respectively. Figure 1 shows that there are three major gene communities in KIRC and LIHC, whereas PRAD shows a higher number of relevant gene clusters. However, all IC genes of LIHC are well-connected among each other, differently from the other two cancer types where some gene clusters are isolated. The average degree of the IC genes in each fused network is 261.44 for KIRC, 403.82 for LIHC, and 103.91 for PRAD; as expected, it is much higher than the one of the entire network. This confirms the validity of the modularity method presented in “Network fusion for gene extraction” section, which finds well-connected groups of genes in the fused networks.

**Fig. 2:**
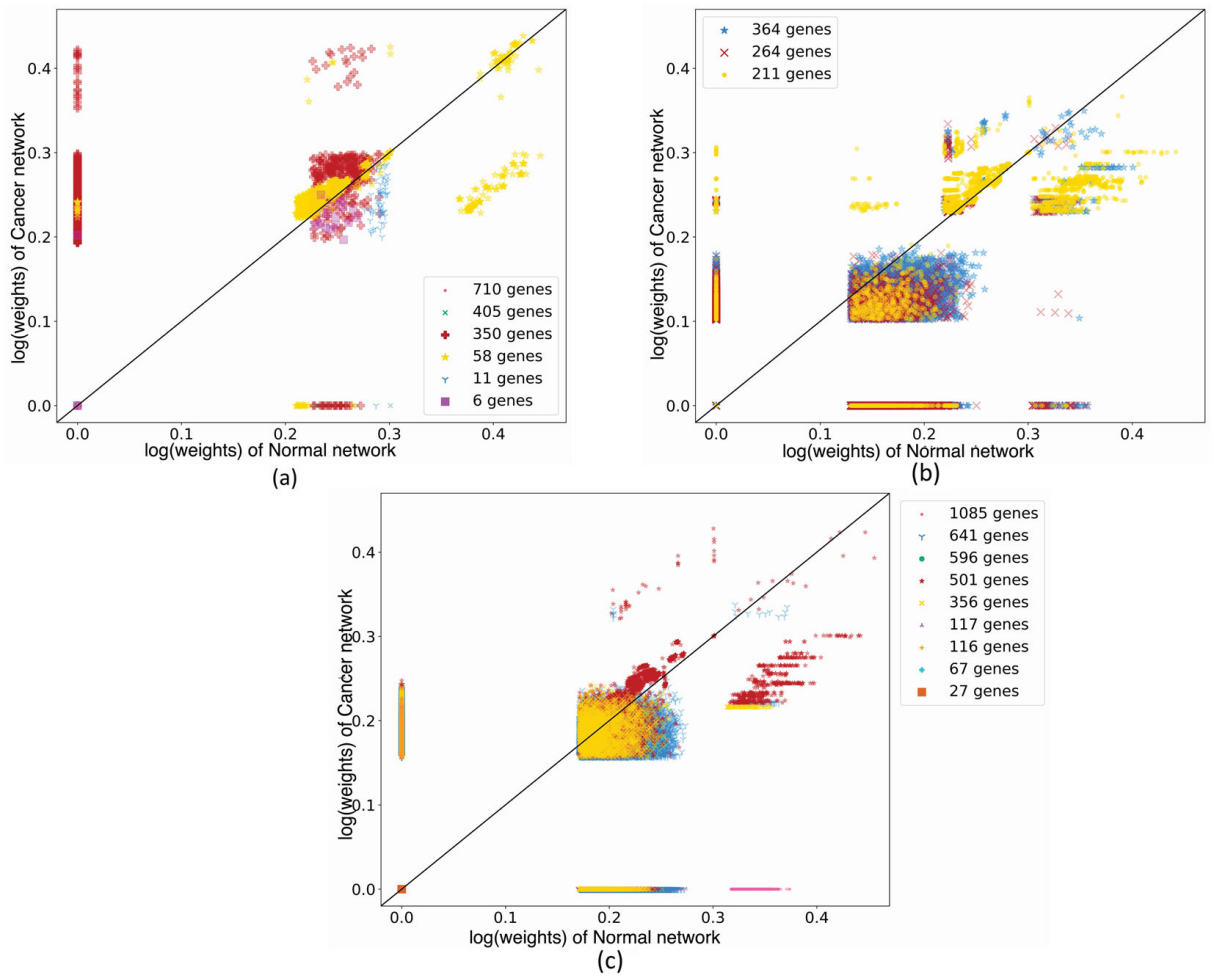
Gene pair relationships. **a** KIRC, **b** LIHC, and **c** PRAD IC gene pair relationships (edge weights) in Normal vs. Cancer co-expression networks, based on both Euclidean distance and Pearson’s correlation of gene expression values. Different colors and symbols represent different gene communities; symbol color transparency shows the amount of IC gene pairs that a point represents.

Figure 2 illustrates the scatter plots of IC gene pair relationships, based on both Euclidean distance and Pearson’s correlation of the gene expression values in Normal and Cancer co-expression networks. In these plots, a point represents the edge between a pair of IC genes, and the point values on the *x* and *y* axes represent the edge weights in the Normal and Cancer network, respectively. Each gene community is displayed with a specific symbol and color, and its cardinality is shown in the plot legend. Moreover, the transparency of the symbol of a point in a plot shows the number of edges that the point represents: the more colored is the symbol, the more are the edges that the point represents. In the scatter plots in Fig. 2, overall the points are either near the plot diagonal, showing similarity among the normal and cancer conditions, or on the plot axes, showing differences in the relationship between the expression values of the same gene pair in the two conditions. This indicates that the IC genes have either very similar or very different expression relationships in the two conditions, proving the validity of the adoption of the gene Similarity Network Fusion method illustrated in “Network fusion for gene extraction” section.

### Computational evaluation of the IC genes

We start the computational assessment of our pipeline for cancer biomarker identification by evaluating the ability of the identified IC genes in discriminating between cancer and normal samples. According to the TCGA data sets, each sample belongs to one category, either “Primary Solid Tumor” or “Solid Tissue Normal”. First, we apply the LASSO regression analysis on the IC genes using their expression profiles as features, thus extracting a smaller set of relevant genes (which we call IC_*L*_ genes) for each cancer type; these genes can be used for better normal/cancer classifications. The gene expression profiles of the IC_*L*_ genes are given as input features to the Random Forest Classifier so as it can classify the samples according to their types, i.e., tumoral or normal. Table 3 contains the total number of IC_*L*_ genes and the number of microRNAs present in each IC_*L*_ gene set of the considered cancer types. We also demonstrated the independence of the identified IC genes from the number of considered samples (Supplementary Material Section Robustness of IC genes using different numbers of LIHC cancer samples).

**Table 3:** Number of RNA genes and miRNAs in the fused networks.

Gene set	KIRC	LIHC	PRAD
All	14,189 (1397)	13,016 (1421)	13,508 (1411)
All_*L*_	1104 (242)	188 (112)	122 (81)
IC	1543 (727)	839 (57)	3502 (125)
IC_*L*_	139 (92)	108 (49)	95 (63)
EuclIC_*L*_	117 (0)	133 (0)	102 (0)
PearsIC_*L*_	112 (27)	147 (0)	70 (0)
DE	1059 (54)	434 (27)	218 (24)
DE_*L*_	20 (18)	20 (15)	15 (9)
IC ∩ DE	57 (35)	30 (2)	67 (8)
IC ∩ DE_*L*_	2 (12)	1 (2)	4 (7)
IC_*L*_ ∩ DE	6 (11)	4 (2)	2 (6)
IC_*L*_ ∩ DE_*L*_	0 (10)	0 (2)	0 (6)
All_*L*_ ∩ IC	196 (129)	88 (45)	80 (60)
All_*L*_ ∩ IC_*L*_	128 (89)	83 (43)	75 (55)
All_*L*_ ∩ DE	165 (27)	10 (9)	6 (7)
All_*L*_ ∩ DE_*L*_	3 (18)	0 (8)	0 (6)

Number of total genes (and miRNA ones only) in the fused networks, in the extracted gene sets, and shared between gene sets. All: fused network, IC: Integrated community, and DE: Differentially expressed genes; All_*L*_, IC_*L*_, EuclIC_*L*_, PearsIC_*L*_, DE_*L*_: LASSO reduced gene sets (EuclIC_*L*_ and PearsIC_*L*_ are from the IC genes identified in the single-type Euclidean distance and Pearson’s correlation networks, respectively).

**Fig. 3:**
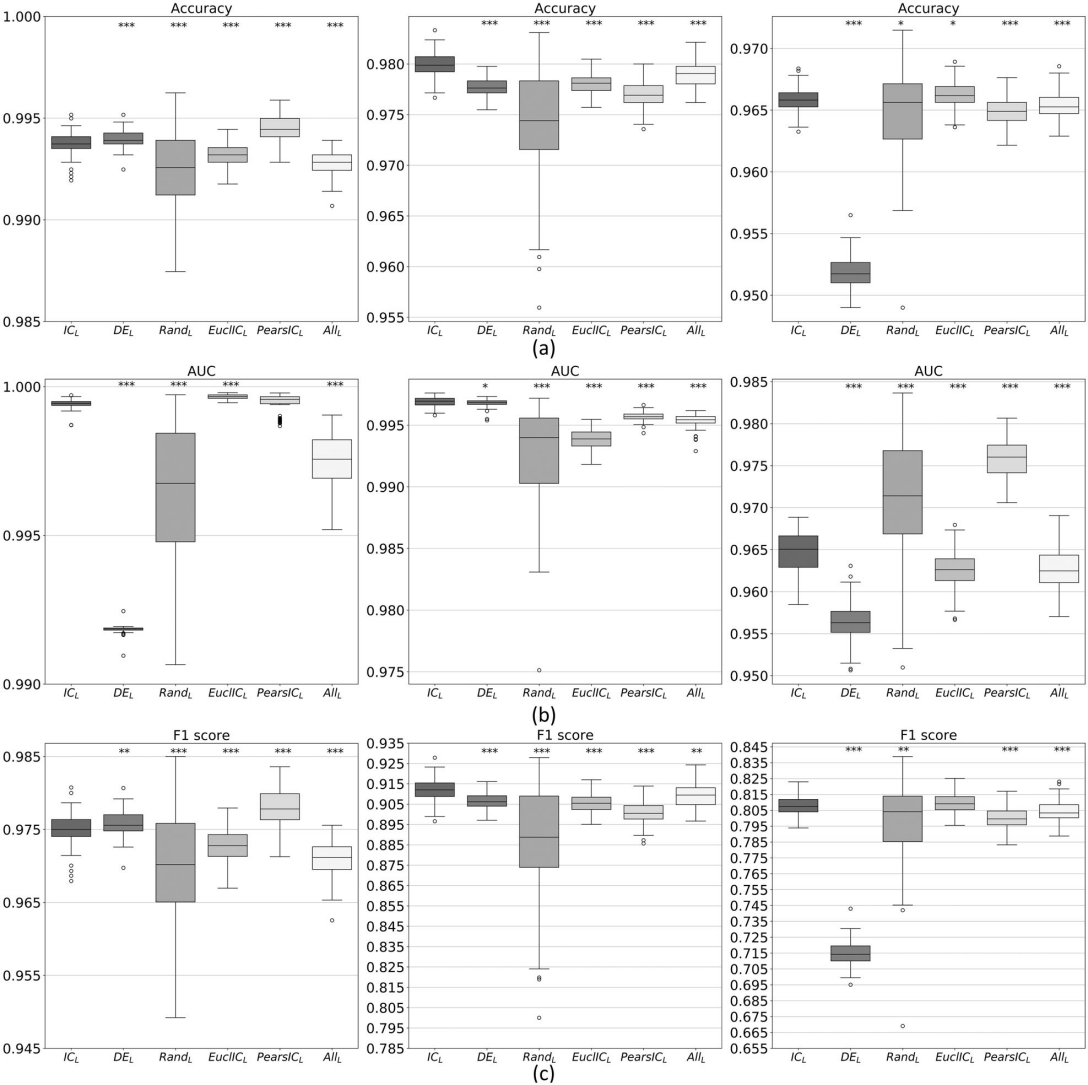
Boxplot of the performances of the classification. Boxplots of the Accuracy, AUC, and F1 score metrics for the normal/cancer classification of the three cancer types: **a** KIRC, **b** LIHC, and **c** PRAD. Each box in a plot represents the performance of a different gene set; IC_*L*_: Integrated community, DE_*L*_: Differentially expressed, Rand_*L*_: Randomly selected, EuclIC_*L*_: Euclidean distance network IC, PearsIC_*L*_: Pearson’s correlation network IC, and All_*L*_: fused network all genes selected with LASSO. ***, **, and * indicate significant difference from IC_*L*_ with *p*-value 0.001, 0.01, or 0.05, respectively.

We perform the classification one hundred times and each time we compute the classification performance of the IC_*L*_ genes. We repeat the same classification using the other five gene sets and compare their performances with the IC_*L*_ gene ones, reporting the result boxplots in Fig. 3. The other evaluated gene sets include DE_*L*_ genes, which LASSO extracts from the differentially expressed gene set, Rand_*L*_ genes, extracted by LASSO from a random set of the network genes with the same IC gene cardinality, EuclIC_*L*_, and PearsIC_*L*_ gene sets, which LASSO extracts from the IC genes identified in the single-type Euclidean distance or Pearson’s correlation similarity network, respectively, and All_*L*_ genes, extracted with LASSO from all the nodes of our networks. The performance comparison among these gene sets enables a thorough evaluation of our method since Rand_*L*_ and All_*L*_ represent the computational baselines, EuclIC_*L*_ and PearsIC_*L*_ represent the single contribution of each gene similarity network used in our approach, and DE_*L*_ is the biologically relevant reference. We also compute the Wilcoxon signed-rank test between the performance metrics of the IC_*L*_ gene set and of each other gene set to evaluate the statistical significance of their difference, reporting the results in Fig. 3.

All the IC_*L*_ boxplots are significantly different from the others, except the EuclIC_*L*_ F1 score one in PRAD (yet, IC_*L*_ genes outperform the EuclIC_*L*_ ones in all the other considered cases and tumors, except in PRAD Accuracy and in KIRC AUC where their performance is slightly lower). Compared to DE_*L*_ genes, IC_*L*_ genes have overall similar performances in KIRC (except their much better AUC) and in LIHC, but much better performances in PRAD. With respect to PearsIC_*L*_ genes, IC_*L*_ genes have the same performance in KIRC AUC and lower performance slightly in KIRC Accuracy and F1 score and more markedly in PRAD AUC, but they have better performances in all the other cases. Finally, IC_*L*_ genes have always better performances than the baseline All_*L*_ and Rand_*L*_ genes, except for the PRAD AUC of the latter ones, which however have always a very wide variance as expected.

In Table 3, we report the cardinalities of the All, IC, and DE gene sets and of their corresponding LASSO reduced sets, together with the cardinalities of the intersections among these different sets. Table 3 highlights that IC and IC_*L*_ gene sets only slightly overlap the DE gene set. This proves that our pipeline allows identifying genes that classify well the two normal and cancer conditions without being differentially expressed. Thus, the IC_*L*_ genes are particularly interesting since, although their expression does not vary significantly across conditions, they do have relevant gene expression network interactions, an aspect that is not considered in the differential expression analysis.

### Hazard ratio comparison between IC and DE genes

To further evaluate the clinical relevance of IC genes, we compute their hazard ratios for each cancer type, and we compare the resulted survival curves with the ones calculated for the DE genes. For this analysis, we use the patient metadata available in the TCGA repository, specifically the patient vital status and survival (days to death and days to follow-up) as the event and the time variable, respectively, of the Cox proportional-hazards model. The expression profile of each IC gene in the cancer samples of all patients for each cancer type is used one by one as a feature variable of the univariate Cox proportional-hazards model, which gives as output the hazard ratio and *p*-value of the gene for the survival estimation. Then, we select only the genes that are statistically significantly associated with the overall patient survival, i.e., those with *p*-value smaller than 0.05 after multiple testing correction using the false discovery rate (FDR) procedure^20^. Moreover, we remove those genes that have a hazard ratio between 0.9 and 1.1, since they do not clearly indicate a negative or positive association with the event probability. Conversely, genes with a hazard ratio smaller than 0.9 have expression levels directly proportional to the goodness of the clinical outcome, and genes with a hazard ratio greater than 1.1 have higher expression values associated with worse clinical outcomes^21^.

**Fig. 4:**
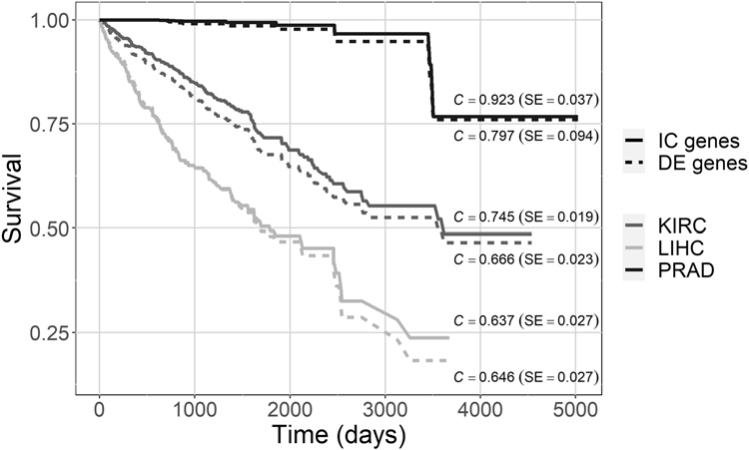
Survival curves. Survival curves of multivariate Cox proportional-hazards models with relevant IC (full line) or DE (dashed line) genes for each cancer type. The curves show the probability of survival over time (in days) for the considered data sets. C Concordance statistic, SE standard error.

The univariate Cox proportional-hazards model assessment of the prognostic power of each IC gene identifies 38, 1, and 5 genes for KIRC, LIHC, and PRAD, respectively, whereas the same analysis for each DE gene yields only 4, 7, and 2 genes, respectively. The higher number of IC genes significantly associated with the survival event probability (for both KIRC and PRAD cancer types) compared to one of the DE genes indicates that the IC genes are more important in terms of survival analysis than the DE ones. To visually compare these results, we use each group of the significant IC and DE genes from the univariate analysis to build a multivariate Cox proportional-hazards model for each cancer and gene type (IC or DE). In Fig. 4, the survival curve of each multivariate model is represented with its Concordance statistic (C) and standard error (SE); the former one is a well-known measure of goodness-of-fit in survival models, ranging from 0 to 1 with 0.5 denoting random fit equivalence, values over 0.7 representing good model fit, and 1 indicating best fit^22^. The model built with the relevant IC genes as variables gives high and much better Concordance statistic than the one built with the relevant DE genes for PRAD and KIRC (C equal to 0.923 vs. 0.797 and 0.745 vs. 0.666, respectively), meaning that the two relevant IC gene sets may have interesting biological qualities, to be experimentally evaluated. Moreover, the differences between the DE and IC survival curves are statistically relevant for KIRC and PRAD (*p*-value 1.55 × 10^−3^ and 9.41 × 10^−3^, respectively). Instead, for LIHC the two survival curves are very similar (C equal to 0.637 vs. 0.646, respectively).

### Knowledge-based evaluation

To complete the assessment of the IC gene set of each cancer type, we perform three different knowledge-based evaluations. First, we evaluate the proportion of IC genes known to be associated with the considered tumor in the literature, then we check if the IC genes of each cancer type are known to be druggable, and finally we perform a gene set enrichment analysis to evaluate the biological functions of the IC gene sets.

We evaluate the significance of the IC-gene/cancer-type association through a systematic literature evaluation. From PubMed (https://www.ncbi.nlm.nih.gov/pubmed/), we automatically retrieve all scientific publications that report both the gene symbol and the related cancer type, and we perform a Fisher’s exact test^23^ for the enrichment of the genes associated with the related cancer type in at least one publication within the IC gene subset versus all genes in the cancer-type fused network. The results show a high number of IC genes known to be associated with the related cancer type (340, 385, and 1341 for KIRC, LIHC, and PRAD, respectively), with a very significant statistical enrichment in all considered cancer types (*p*-values 1.19 × 10^−37^, 1.05 × 10^−12^, and 1.25 × 10^−43^, respectively).

Furthermore, we evaluate the amount of drug actionable IC genes with respect to all druggable genes in the same fused network. For each cancer type, we extract the subset of fused network genes that are considered as pharmacologically active targets in DrugBank (https://www.drugbank.ca/releases/latest), i.e., that are directly related to the mechanism of action of at least one drug. We perform the Fisher’s exact test for the enrichment of such genes in the IC gene set, identifying a significant enrichment for KIRC and LIHC (*p*-value 8.10 × 10^−4^ and 2.23 × 10^−3^). PRAD IC gene set does not result significantly enriched for drug actionable targets; yet, it contains 115 hits and 4 of them (*ACPP*, *MAP1A*, *VKORC1*, and *VKORC1L1*) are targets of drugs used for the PRAD treatment. This evaluation clearly shows that several IC genes of each cancer type are already known as druggable, indicating those of them that are targets of drugs not yet used for the specific cancer-type treatment as priorities for effective drug repurposing. Furthermore, it suggests the chance that other IC genes not yet annotated as pharmacologically active targets may be drug actionable and possible targets for new cancer drug treatments.

Finally, we perform the functional analysis of the three identified sets of IC genes using g:Profiler (https://biit.cs.ut.ee/gprofiler/gost), a Web server for functional enrichment analysis on a huge variety of data sources, revealing if the IC genes are related to important biological processes for each cancer type. KIRC IC genes are significantly enriched in some interesting Gene Ontology Biological Processes (GO:BP) and Kyoto Encyclopedia of Genes and Genomes (KEGG) pathways, including gene silencing by miRNA, sprouting angiogenesis, vasculature development, cell migration, and microRNAs in cancer. Indeed, angiogenesis is a fundamental process for cell proliferation in KIRC as reported in KEGG (https://www.kegg.jp/kegg-bin/show_pathway?hsa05211), where the *PDGFBA, TGFB1, TGFB2, TGFB3*, and *VEGF* genes are known to be involved in the development of new blood vessels. KIRC IC genes significantly enriched in angiogenesis are 27 (including 26 miRNAs). Interestingly, among them: DLL1 gene product interacts with proteins of the *VEGF signaling pathway*, while *hsa-mir-7, hsa-mir-29c, hsa-mir-125a, hsa-mir-126, hsa-mir-296, hsa-mir-361, hsa-mir-424, hsa-mir-495,* and *hsa-mir-503* miRNAs target VEGFA gene, as reported in miRTarBase (http://mirtarbase.mbc.nctu.edu.tw); instead, *hsa-mir-10a, hsa-mir-23b*, and *hsa-mir-375* are known to regulate the genes involved in the *TGF-*β signaling pathway (TGFB1, TGFB2, and TGFB3), whereas *hsa-let-7b, hsa-let-7f*, and *hsa-mir-146b* target *PDGFB* gene. Thus, the majority of KIRC IC genes involved in angiogenesis is also involved in the main pathways of the KIRC disease. Moreover, the *PAK1* and *PIK3R1* genes have a crucial role in cell migration and mobility in the KEGG renal cell carcinoma pathway, and both of them have been extracted by our pipeline as KIRC IC genes.

Regarding LIHC IC genes, they are significantly enriched in several GO:BP particularly relevant for LIHC, including oxidation-reduction process, organic acid catabolic process, drug metabolic process, fatty acid metabolic process, and lipid metabolic process. Indeed, KEGG reports fatty acid oxidation/biosynthesis, adipocytokine signaling pathway, and calcium signaling pathway as important pathways for cell proliferation and survival in the LIHC disease (https://www.kegg.jp/kegg-bin/show_pathway?hsa05225). Furthermore, several of the LIHC IC genes, including *EGFR, GSTA1, GSTO1, MGST1, MGST2, PIK3K1,* and *TXNRD2*, are also known to be involved in LIHC disease pathways.

PRAD IC genes are instead significantly enriched in GO:BP metabolic process and regulation of gene expression. Among PRAD IC genes known to be involved in the KEGG prostate cancer disease pathway (https://www.kegg.jp/kegg-bin/show_pathway?hsa05215), *BAD, CREB1, CREB3L4, CREBBP, EP300,* and *TCF7L1* genes are all involved also in the metabolic pathway known to regulate cell apoptosis and proliferation, while *GSTP1* regulates the carcinogenesis of PRAD and it is also involved in the metabolic process; instead, *SPINT1* and *ZEB1* regulate gene expression and they also play an important role in the PRAD pathway by promoting cellular migration and invasion.

## Discussion

The aim of this study is the extraction of relevant cancer gene biomarkers through the innovative integration of multiple gene co-expression networks, as proposed in “Building of gene co-expression networks” section, and the fusion of normal and cancer condition networks, described in “Network fusion for gene extraction” section. In order to do so, we compute the Euclidean distance and Pearson’s correlation similarity measures between the expression profiles of each pair of genes for the normal and cancer data sets of each cancer type considered. Then, the so-built adjacency matrices of the Euclidean distance and Pearson’s correlation co-expression networks are normalized and summed together to obtain, for each condition, an integrated network that represents all the relevant characteristics highlighted by the two measures. The final cancer-type-specific networks are obtained by fusing the two condition-specific networks with the SNF algorithm.

IC genes, extracted from each fused network, are of great interest from the cancer type perspective. They give very good normal/cancer sample classifications, even better than the ones provided by cancer biomarker genes identified with the classical differential expression analysis, as reported in Fig. 3. Comparisons show also that the integration of multiple co-expression networks outperforms the single-type co-expression analysis. IC genes exhibit interesting results also in terms of survival analysis: 44 of all IC genes are significantly associated with the survival event probability, a relevantly higher number than the 6 differentially expressed genes significantly associated with survival. Moreover, the multivariate Cox proportional-hazards model of the significant IC genes for each cancer type gives a better Concordance statistic, i.e., a better fit, than the model built by using the differentially expressed genes as variables. Finally, the three-fold knowledge-based evaluation proved that IC genes may be potentially valuable cancer biomarkers, possibly actionable for drug treatments, significantly enriched in the main pathways of the disease, and including several genes known to be of interest for the specific cancer type.

IC genes that may be novel cancer biomarkers are those not associated with the cancer type in PubMed and that are actionable (i.e., there are drugs that can target them). In particular, for KIRC disease, *MT-CYB, NDUFV3, PARP3*, and *TOP1MT* gene products are labeled as actionable in DrugBank and they may have an important role in the regulation of the MAPK signaling pathway, and consequently of cell proliferation. Indeed, these gene products are all indirectly associated with KRAS or HRAS (two of the main genes belonging to the MAPK signaling pathway) by means of another protein (as reported in BioGRID - https://thebiogrid.org). Moreover, *hsa-mir-7, hsa-mir-29c, hsa-mir-125a, hsa-mir-296, hsa-mir-361, hsa-mir-424, hsa-mir-495*, and *hsa-mir-503* are interesting miRNAs because they regulate two of the fundamental genes of the VEGF signaling pathway. Also, *hsa-mir-23b* and *hsa-mir-375* regulate genes in the *TCF*-β signaling pathway, another crucial pathway in KIRC, and *hsa-let-7b*, *hsa-let-7f*, and *hsa-mir-146b* can target *PDGFB*, which is a well-known oncogene^24^. However, PubMed does not contain evidence of the involvement of these genes in KIRC disease; thus, they are good candidate biomarkers for experimental investigation according to our findings.

For LIHC disease, we found 13 actionable IC genes that have not yet been studied for this disease. Among them, the *FGB* gene encodes for the beta component of the fibrinogen, a glycoprotein that regulates cell adhesion and spreading. Interestingly, according to BioGRID, the *FGB* gene product directly interacts with the *PI3K* enzyme family, which has an important role in the LIHC cell survival pathway. Moreover, *ABAT, ETFDH, F7*, *QPRT,* and *RAMP1* gene products are druggable and they all indirectly interact with the *PI3K* enzyme family by means of another protein; thus, a deeper examination of their interactions could give important insights for LIHC disease. There are also IC miRNAs, neglected by the literature for their association with LIHC disease, that target the PI3K enzyme family, such as *hsa-mir-10b, hsa-mir-30a, hsa-mir-93, hsa-mir-126, hsa-mir-143,* and *hsa-mir-375*.

Actionable PRAD IC genes, whose involvement in PRAD disease is not yet annotated, are 24. Among them, *ACAA1*, *GART, PDE9A, RPL3, TUBA1A,* and *TUBG1* gene products interact with several proteins known to be involved in the PRAD pathway and particularly important for apoptosis inhibition and tumor growth. Thus, they could be possible PRAD biomarkers. Moreover, there are several IC miRNAs not yet studied for PRAD that target pivotal genes involved in the PRAD pathway, and that can also affect the metabolic process of the disease; they are *hsa-let-7b, hsa-mir-23b, hsa-mir-26a, hsa-mir-26b, hsa-mir-30a, hsa-mir-101, hsa-mir-193b,* and *hsa-mir-199a*.

All mentioned IC genes seem to be cancer-type-specific, i.e., they are uniquely extracted from a single cancer-type-specific network. However, among all IC gene sets there are three common miRNAs worth mentioning due to their cancer-related properties: *hsa-let-7b, hsa-mir-23b* and *hsa-mir-375*.

All these findings prove the importance of the use of co-expression networks and the relevance of the integration of different similarity measures that we developed. They allow a finer identification of genes (the IC ones) that, thanks to their relations in the fused co-expression networks built, provide better normal/cancer sample classification performance than the DE genes, which are more commonly used for this task.

Moreover, our pipeline is easy to be extended by, for example, considering mutation signatures. The similarities among mutation samples could be combined together with the co-expression networks using the SNF method. In this way, the fused networks could identify IC genes whose expression profiles and mutation signatures are either highly similar or very different between the normal and cancer condition.

## Methods

**Fig. 5:**
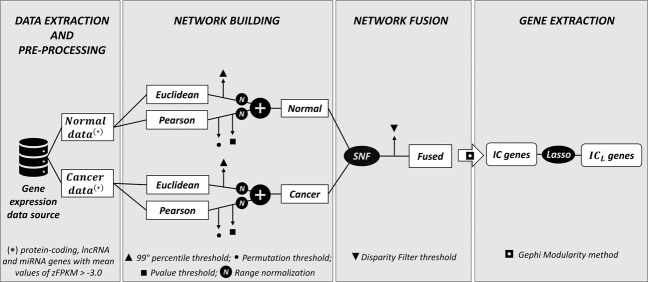
Workflow representation of the pipeline. Defined workflow for the identification of candidate cancer gene biomarkers.

This section describes our novel multi-step methodology to identify candidate cancer gene biomarkers, which is schematically illustrated in Fig. 5. After the extraction and pre-processing of gene expression data from normal and cancer conditions (Data Extraction and Pre-Processing panel in Fig. 5), two gene similarity measures are computed and used for the construction of different gene co-expression networks, which are then combined to have one normal sample network and one cancer sample network for each cancer type considered (Network Building panel in Fig. 5). Finally, the two condition-specific networks of each cancer type are fused (Network Fusion panel in Fig. 5) in order to extract relevant gene biomarkers for cancer diagnosis (Gene Extraction panel in Fig. 5).

### Data extraction and pre-processing

To infer gene co-expression networks, we extract RNA-Seq and miRNA-Seq data sets for the human GRCh38 assembly from the TCGA repository^17^ using the GenoMetric Query Language (GMQL), an innovative query language for genomic data and metadata^25^. The first data set contains gene expression quantifications, i.e., the number of reads that map to each gene using the RNA-Seq technique^26^, whereas the second data set contains microRNA (miRNA) quantifications derived from the next-generation sequencing of microRNAs^27^. Specifically, we consider the data of three representative tumors: kidney clear cell carcinoma (KIRC), liver hepatocellular carcinoma (LIHC), and prostate adenocarcinoma (PRAD). TCGA data sets of these cancer types have a suitable number of both normal and cancer patients’ samples with both RNA-Seq and miRNA-Seq data (Table 4), with a balanced ratio of samples between the two conditions (i.e, whose number of samples is balanced between the normal and cancer condition). Furthermore, all these samples have valuable metadata describing patients’ vital status and survival (days to death and to follow-up).

**Table 4:** Samples.

Samples	KIRC	LIHC	PRAD
Cancer	487	370	495
Normal	71	50	52
Total	558	420	547

Number of TCGA patients’ samples considered.

We organize the extracted data in the form of matrices that have as elements the values of fragments per kilobase of transcript per million mapped reads (FPKM) for each gene/sample pair, considering only the patients with samples in both the two RNA-Seq and miRNA-Seq data sets. Due to the presence of measurement errors and noisy genes, from RNA-Seq data sets we get only protein-coding and long non-coding genes; instead, from miRNA-Seq data sets, we obtain microRNA gene quantifications. Additionally, we use the normalization method and threshold reported in Hart et al.^28^ to distinguish between biologically relevant genes and noisy ones (by genes we refer to both RNAs and microRNAs). The selected RNA-Seq data have a mean value of normalized fragments per kilobase of transcript per million mapped reads (zFPKM) higher than −3.0 in both normal and cancer conditions^28^. zFPKMs are *z*-scores of log(FPKMs), and the threshold equal to −3.0 accurately determines which are the active genes and which the background ones^28^.

### Building of gene co-expression networks

The process we propose for constructing gene co-expression networks can be split into three main steps: computation of similarity measures between genes, statistical thresholding to reduce over-connectivity of the networks, and integration of the similarity measures for each condition, normal or cancer, for each cancer type.

In a gene co-expression network, the nodes correspond to genes and the edges represent the relations between the gene expression profiles found with a not null similarity measure^4^. There is a huge variety of similarity measures that can be used; each one quantifies the degree of association between expression profiles by representing different characteristics^29^. Here, we consider two types of networks, built by employing two different similarity measures: one the Euclidean distance and the other the Pearson’s correlation. The first metric identifies similar or identical expression regulations having a positive linear correlation, and it is influenced by the magnitude of changes in the expression profiles^30^. Whereas the second one reflects the degree of linear correlation between two patterns of expression^29^. Since the two conditions, normal and cancer, are maintained separate, for each condition and for each cancer type, we build one Euclidean distance network to highlight linear similarity and one Pearson’s correlation network to highlight pattern similarity^30^ between gene expression profiles. However, the so-created networks in this first step have a huge number of edges with not null weights.

The over-connectivity of the networks does not allow highlighting the relevant associations among genes. We deal with this issue in the second step, by removing all the network edges that can be considered pointless for our purpose. To do so, we use statistical thresholding methods on the weight of the edges^31^. Regarding the Euclidean distance networks, we keep only the edges with a weight higher than the 99th percentile of the distribution of the network edge weights. This allows us to consider only pairs of genes that have a high difference in expression. For the Pearson’s correlation networks instead, we perform a permutation test that allows detecting a significant threshold for the Pearson’s correlation values^31^. By randomly shuffling 10 times the expression data, we compute the average permuted distribution of Pearson’s correlation values. Then, we identify its lower and higher limit values as the significant thresholds, filtering out the network edges with a Pearson’s correlation value between these thresholds^32^. We also evaluated the robustness of the permutation test by considering increasing numbers of times shuffling in the Supplementary Material Section Permutation tests on LIHC Normal and Cancer networks. Since the networks created with Pearson’s correlation are very dense, we use the computed *p*-value of Pearson’s statistic to further prune the network edges. We sort these *p*-values and we consider as a threshold their percentile that allows having a number of edges similar to the one of the corresponding Euclidean distance network. This is needed to have the two metrics balanced while at the same time resolving the over-connectivity problem of the co-expression networks.

The last step is the union of the two measure networks in order to have a single network for each condition. The final networks highlight the pairs of genes in each condition, normal or cancer, that have expression profiles highly different (based on their Euclidean distance) and/or with a similar pattern (based on their Pearson’s correlation). In order to do so, since any network is described by the adjacency matrix of its edge weights, first, we normalize the logarithm values of the Euclidean distance adjacency matrices, by dividing them by the maximum logarithmic value in the matrix, and we compute the absolute values of the Pearson’s correlation adjacency matrices. Then, we sum the two obtained matrices for each condition, so to get one adjacency matrix describing the normal sample network and one the cancer sample network. Each condition-specific network contains all the meaningful characteristics highlighted by the two similarity measures considered.

### Network fusion for gene extraction

Network Fusion panel in Fig. 5 shows the final processing to fuse normal and cancer sample networks; we do so by using the Similarity Network Fusion (SNF) algorithm^33^, which can fuse different similarity networks (in this study, normal and cancer co-expression networks) highlighting the presence of groups of connected genes in the resulting combined network^34^. It has been used to integrate different genomic data types of the same patients^33,34^, for emphasizing clusters in patient subtyping^35^, and for predicting biological associations^36,37^.

The SNF algorithm consists of two important steps^33^; here, we briefly describe them reporting the main formulas of this method. Our normal and the cancer sample networks are obtained by combining the gene co-expression networks of two similarity measures (Euclidean distance and Pearson’s correlation) in normal and cancer condition, respectively. The vertices of the two networks correspond to the considered genes {*g*_1_, *g*_2_, …, *g*_*n*_} and the edges are the gene combined similarities. Suppose *W*^(*n*)^ and *W*^(*c*)^ represent the adjacency matrices of the normal and cancer sample networks, respectively, with *W*^(*n*)^(*i*, *j*) and *W*^(*c*)^(*i*, *j*) indicating the similarity between genes *g*_*i*_ and *g*_*j*_ in normal and cancer condition, respectively. The first step of the SNF to compute the fused network is the use of the K nearest neighbors (KNN)^33^ to measure local affinity in each network and to compute the kernel matrices *S*^(*n*)^ and *S*^(*c*)^ for *W*^(*n*)^ and *W*^(*c*)^, respectively, as follows^33^:

1 $$S(i,j)=\left\{\begin{array}{ll}\frac{W(i,j)}{{\sum }_{k\in {N}_{i}}W(i,k)},&j\in {N}_{i}\\ 0&{\mathrm{otherwise}}\end{array}\right.$$

where *S* and *W* are the general kernel matrix and adjacency matrix, respectively, and *N*_*i*_ is the set of neighbors of *g*_*i*_, i.e., the set of genes that are directly connected with *g*_*i*_ in the network. Once the kernel matrices are computed, the second most important step of the SNF is the iteratively update of the similarity matrix *W*^33,36^. Specifically for the fusion of normal and cancer sample networks, we use the following formulas:

2 $${W}_{t+1}^{(n)}={S}^{(n)}\times {W}_{t}^{(c)}\times {({S}^{(n)})}^{T}$$

3 $${W}_{t+1}^{(c)}={S}^{(c)}\times {W}_{t}^{(n)}\times {({S}^{(c)})}^{T}$$

where ${W}_{t = 0}^{(n)}={W}^{(n)}$ and ${W}_{t = 0}^{(c)}={W}^{(c)}$ represent the initial two matrices, at iteration *t* = 0, for the normal and cancer condition, respectively. At each iteration, the matrices *W*^(*n*)^ and *W*^(*c*)^ are updated by generating two parallel intertwined diffusion processes^33^. The final fused adjacency matrix is computed after t* iterations as follows^33,36^:

4 $${W}^{({\mathrm{fused}})}=\frac{{W}_{{t}^{* }}^{(n)}+{W}_{{t}^{* }}^{(c)}}{2}$$

where t* is the iteration that leads to the convergence of the algorithm, i.e., when the relative difference of two consecutive iterations ($\frac{\parallel {W}_{t+1}^{({\mathrm{fused}})}-{W}_{t}^{({\mathrm{fused}})}\parallel }{\parallel {W}_{t}^{({\mathrm{fused}})}\parallel }$) is <10^−5^.

The SNF algorithm filters out weak similarities not shared by the input networks, reducing the noise. Instead, depending on local affinities, strong similarities present in one or both networks are preserved, as well as weak similarities supported by both input networks (i.e., small communities of weak/mixed edges that are consistent in both networks)^33^. Therefore, the method has a de-noising function on the input networks, allowing to combine them highlighting groups of nodes with common similarities in the two input networks, or with much higher similarities in one of them^34^. With respect to the single input networks, the fused network gives a much clearer picture of the presence of connected gene clusters^33^.

Finally, the fused network of each studied cancer type is passed through an additional pruning step, called disparity filter^38^; it extracts the backbone structure of an undirected weighted network without destroying the major features of the network^38^. The disparity filter requires a p-value threshold, which identifies the significance of the comparison between a uniform distribution of each node of the network and a null model^38^. We set this threshold at the 99th percentile of the overall *p*-value distribution of each network. Then, gene communities in the fused network are extracted using the modularity method implemented in Gephi^39^, a software that allows representing a network and calculating network measures. The modularity method measures how well a network decomposes into modular communities by counting, up to a multiplicative constant, the number of edges within groups that are not in an equivalent permuted network (where edges are placed at random)^40,41^. Genes in these communities are those selected as relevant in the fused network; we name them Integrated Community (IC) genes.

### Computational validation methods and metrics

For the validation of the gene sets that our pipeline extracts we use several computational methods. First, we use the LASSO regression method^42^ (“LASSO regression” section) for the feature selection from the gene sets found in “Network fusion for gene extraction” section and from the differentially expressed genes (“Differential gene expression analysis” section) and other gene sets for reference comparison, whose performances in classifying cancer versus normal samples are subsequently compared and evaluated using a set of performance metrics (“Sample classification and performance metrics” section). Moreover, we evaluate the clinical relevance of the extracted gene sets by means of survival analysis^43^, performed through the Cox proportional-hazards model^44^ (“Cox proportional-hazards model” section).

#### LASSO regression

The Least Absolute Shrinkage and Selection Operator (LASSO) is a regression method commonly used for feature selection; it allows to obtain better prediction accuracy and interpretability than standard regression models by shrinkage of feature coefficients and by providing a sparse solution, i.e., with some feature coefficients null^42^. Thus, we use LASSO to select representative genes within the communities extracted from the fused network, which we name IC_*L*_ genes, as well as within other gene sets; this allows us to have much smaller relevant gene sets, which can be better used for normal/cancer classification.

The basic form of LASSO regression was originally introduced in the context of least squares^42^. Let (*y*_1_, *x*_1_),…, (*y*_*p*_, *x*_*p*_) be *p* output/input pairs and consider a linear regression model as follows:

5 $${y}_{i}={\beta }_{0}+{\beta }_{1}{x}_{i1}+\cdots+{\beta }_{p}{x}_{ip}+{\epsilon }_{i}$$

where *ϵ*_*i*_ are random quantities with mean zero^42^. The objective of LASSO is to find the *β* values that minimize the empirical risk for a given loss function, expressed as follows^42^:

6 $$\mathop{\sum }\limits_{i=1}^{N}{\left({y}_{i}-{\beta }_{0}-\mathop{\sum }\limits_{j = 1}^{p}{\beta }_{j}{x}_{ij}\right)}^{2}$$

subject to $\mathop{\sum }\nolimits_{j = 1}^{p}| {\beta }_{j}| \le t$, where N is the number of observations and *t* is a tuning parameter that defines the amount of regularization. The smaller the value of *t*, the greater the amount of shrinkage towards zero of the coefficients; this leads to obtaining a valuable feature selection, i.e., a sparse subset of variables with non-zero regression coefficients.

#### Differential gene expression analysis

To compare our selected gene sets with a baseline set of reference genes, we perform a differential gene expression analysis to find baseline reference significant genes for each cancer type. This analysis is a well-known method to extract altered expression genes that might be involved in cancer development and that might serve as specific biomarkers for diagnosis^45^. We leverage the *DESeq2* R/Bioconductor package to identify the differentially expressed (DE) genes^46^. DESeq2 uses shrinkage estimators for the dispersion and the expression fold change between the normal and cancer conditions, which is the ratio between the expression mean values of cancer over the normal samples. We obtain the DE genes by selecting those that have an adjusted *p*-value (i.e., after multiple testing correction using the Benjamini and Hochberg method) lower than 0.05 and an absolute value of the log2 fold change greater than 2.0.

#### Sample classification and performance metrics

To evaluate the ability of the considered gene sets in discriminating between cancer and normal sample conditions, we use a Random Forest classifier based on 5-fold stratified cross validation^47^, and three metrics for classification performance evaluation, namely, Accuracy, Area Under the Curve (AUC) and F1 score^48^. The Accuracy is the proportion of true results, either true positive or true negative, in a result population. It measures the degree of correctness of a test on a condition, as given by:

7 $${\mathrm{Accuracy}}=\frac{TP+TN}{N}$$

where *TP* are the true positives, *TN* are the true negatives and *N* is the population cardinality. The AUC is the area under the Receiver Operating Characteristic (ROC) curve. It is a graphic representation of the relationship between the Sensitivity, or Recall, and Specificity of a binary evaluation, defined as follow:

8 $${\mathrm{Sensitivity/Recall}}=\frac{TP}{TP+FN};\quad {\mathrm{Specificity}}=\frac{TN}{TN+FP}$$

where *FN* are the false negatives and *FP* are the false positives of the evaluation. The F1 score is the harmonic mean of Precision and Recall, specified as follow:

9 $${\mathrm{Precision}}=\frac{TP}{TP+FP};\quad {\mathrm{F1}}\,{\mathrm{score}}=2\times \frac{{\mathrm{Precision}}\times {\mathrm{Recall}}}{{\mathrm{Precision}}+{\mathrm{Recall}}}$$

#### Cox proportional-hazards model

Survival analysis is the evaluation of the time to an event of interest (usually the time from diagnosis to death)^43^. We perform it by means of the Cox proportional-hazards model^44^, a regression model commonly used in medical research for testing the association between the survival time of patients and one (univariate) or more (multivariate) predictor variables. We use the hazard ratio statistics instead of the Kaplan–Meier statistics, which is usually used for survival analysis, due to the inability of the latter one to work properly with quantitative variables such as the gene expression values. The Cox proportional-hazards model gives an estimation of how much the specified variables (e.g., the gene expressions) influence the rate of a particular event (e.g., death), called the hazard rate^44^. The hazard function *h*(*t*) denotes the Cox proportional-hazards model and represents the risk of an event (e.g., of dying) at time *t*. Suppose (*x*_1_, *x*_2_, …, *x*_*n*_) indicates a set of *n* variables that determine *h*(*t*) at a certain (survival) time *t*, then the hazard function can be written as follow^44^:

10 $$h(t)={h}_{0}(t)\times {\mathrm{exp}}({b}_{1}{x}_{1}+{b}_{2}{x}_{2}+\cdots+{b}_{n}{x}_{n})$$

where the coefficients (*b*_1_, *b*_2_, …, *b*_*n*_) measure the influence of the variables, *h*_0_ is the hazard baseline and *exp*(*b*_*i*_) is the hazard ratios, i.e., the Cox regression coefficients for the gene expression measurements of normal and cancer samples. A hazard ratio greater than 1 indicates a variable (or a group of variables in the multivariate case) that is positively associated with the event probability (e.g., death), whereas a variable with a hazard ratio smaller than 1 is negatively associated with the event probability.

### Reporting summary

Further information on research design is available in the Nature Research Reporting Summary linked to this article.

## Supplementary information

Supplementary Information

Reporting Summary

## Data Availability

At https://github.com/DEIB-GECO/GeneNetFusion, we provide the TCGA LIHC data sets, the LIHC gene co-expression networks, and for all cancer types considered we also provide the list of IC genes extracted from each of the fused networks. Moreover, the gene-to-gene associations of each fused network can be easily computed by following each step described in GitHub.

## Code availability

We used the Python programming language to implement the main steps of our computational method in separate scripts, respectively regarding the data pre-processing, matrix creation, and matrix fusion, as well as the feature selection and cancer classification evaluation, computed after the extraction of the IC genes with Gephi^39^. Furthermore, we created a Jupyter Notebook (https://jupyter.org/) for the easy execution of all the steps of the developed computational pipeline. All developed software and the Jupyter Notebook are open source and publicly available at https://github.com/DEIB-GECO/GeneNetFusion.
