# Supplementary material for: Computational analysis of fused co-expression networks for the identification of candidate cancer gene biomarkers
Source: NPJ Syst Biol Appl. 2021 Mar 12;7:17. doi: 10.1038/s41540-021-00175-9 (PMC7955132; doi:10.1038/s41540-021-00175-9)
Supplement: Supplementary file 1 — Supplementary Information [file 41540_2021_175_MOESM1_ESM.pdf]

Supplementary Material for
Computational analysis of fused co-expression networks for the identification of candidate cancer gene biomarkers

Sara Pidò Gaia Ceddia
Marco Masseroli
Department of Electronics, Information and Bioengineering,
Politecnico di Milano, via Ponzio 34/5, 20133 Milan, Italy

Gene co-expression network inference

Gene co-expression networks are part of biological network inference in systems biology [1]. They have genes as nodes and predicted gene interactions as edges, where the interactions represent the activation/enhancement or repression/reduction of the transcription of one gene by another [2, 3]. The overall structure of gene networks is one of the major factors that characterize the complexity of cellular life, and the disruption of gene interactions may lead to severe diseases [1–4]. Since discovering gene interactions using wet-lab experiments is not feasible for the whole genome, network inference relies on computational methods that take advantage of gene expression data [1]. We can distinguish four major classes of these computational methods, whose output is a network having a set of genes as nodes and weighted edges representing the confidence of real interactions between

genes [1].

The first class of computational methods for gene network inference regards the *similarity* approach. It is based on the computation of similarity measures for each pair of genes. The advantages are speed and scalability; however, the resulted networks do not take into account the causality of the interactions and their direction [1]. This approach can lead to interesting results; for example, the study by Zhang et al. [5] was the first to perform a *Weighted Gene Co-expression Network Analysis (WGCNA)* providing empirical evidence that topological measures of nodes (i.e., genes) are related to their biological significance. Regarding the predictions of the edges in the networks, this approach well detects densely connected parts of the networks, but it gives false positives for the prediction of linear cascades (i.e., nodes that dependently interact with each other) [6]. To deal with false positives, i.e., the over-connectivity in co-expression networks, Care et al. [7] developed the *Parsimonious Gene Correlation Network Analysis (PGCNA)*, a method that allows the comparison of network structures between cancer types. Thus, in contrast with WGCNA and PGCNA, we focus on the integration of two different similarity measures, dealing with over-connectivity using statistical thresholding as presented in main paper Section 2.2.

Regression analysis is another common approach for network inference [1]. By using a linear regression equation we can predict the relationship between two genes based on their expression data. The edges of the inferred networks represent the computed relationships between all pairs of genes. More advanced regression-based algorithms make use of a feature selection step combined with a regression scoring technique, as presented in Haury et al. [8] (Trustful Inference of Gene REgulation with Stability Selection, or TIGRESS algorithm) and in Huynh-Thu et al. [9] (GEne Network Inference with Ensemble of trees, or GENIE3 algorithm). Regression approaches are more computationally expensive than similarity ones, however the inferred networks carry also information about the direction of the interactions [1]. The methods of this class have an overall good accuracy; yet, they do not perform well as far as it regards small motifs of the networks (i.e., the general structure of the networks is predicted correctly, but local interactions between network nodes have a poor accuracy) [6]. Some improvements have been made over the years by considering both linear and non-linear interactions; for example, Singh et al. [10] developed a regression-based

algorithm that leverages pre-defined non-linear interactions.

Bayesian methods are a major class of methods that have long been applied for network inference [11, 12]. The guiding idea of these methods is that the relationship between two genes is expressed by their conditional probability based on their expression profiles. Thus, the inferred networks (or Bayesian networks, in this case) have genes as nodes and conditional probabilities as edge weights. Multiple Bayesian networks can be inferred from a gene expression dataset; thus, the best Bayesian network is the one that maximizes the likelihood estimation of the edge weights [1]. Disadvantages of this approach include the very high computational cost and the poor prediction of loop motifs in the networks [1, 6].

The last major class of network inference methods includes the information theory methods. Entropy measures the uncertainty of a random variable; for the purpose of network inference, it is used to find the mutual information shared by two variables or genes [1]. The mutual information is obtained by comparing the entropy of the joint distribution with the individual entropies of the two variables; thus, it can be seen as a measure of dependency between variables [13]. Inferred networks have genes as nodes and their mutual information as edges. A limitation of these methods is the constrain of using a binning process for continuous expression data, which is an ongoing problem regarding this approach [14]; differently from our approach, this binning can lead to a loss of information for the inferred networks. A famous example for this class of algorithms is the Algorithm for the Reconstruction of Accurate Cellular Networks, or ARACNe [15], where the networks are computed by combining the computation of the mutual information with two different pruning steps. Other similar methods include the ones by Butte et al. [16], Faith et al. [17], and Meyer et al. [18].

Robustness of IC genes using different numbers of LIHC cancer samples

We evaluated the robustness of our approach by sub-sampling an increasing number of samples of the LIHC tumor. Particularly, keeping all the 50 normal samples, which are available in a

limited number, we randomly extracted 50, 100, 200, 300 cancer samples and we applied to each of such sample sets the same pipeline for the identification of the IC genes that we used for all LIHC samples (370 tumoral and 50 normal samples), as reported in the main manuscript. We noticed that the number of gene communities extracted from the networks in each case is similar to the one extracted in our originally evaluated case with all 370 LIHC tumoral samples, i.e., 2 or 3 communities. Moreover, we compared the IC genes extracted from the networks in the different cases with those extracted from the original one. For each case, Supplementary Table 1 shows the number of extracted IC genes and the percentages of such genes in common with the IC genes extracted from the original network. The values displayed in Supplementary Table 1 demonstrate that our pipeline is robust with respect to the number of samples considered for the construction of the networks. Indeed, the percentages of genes, extracted by varying the number of tumoral samples, that are in common with the IC genes extracted from the whole sample set is always greater than 70%, and the number of extracted IC genes varies only between –11.68% and +9.42% with respect to the number of IC genes from the whole sample set.

Supplementary Table 1: Differences among IC genes using a different number of Cancer samples for the LIHC cancer type.

N^o **cancer samples**	**% common IC genes with whole set**	N^o **IC genes**	**% difference** N^o **IC genes with whole set**	N^o **communities**
50	74%	918	9.42%	2
100	82%	907	8.10%	2
200	78%	787	-6.20%	3
300	82%	741	-11.68%	3
370	100%	839	0%	3

Permutation tests on LIHC Normal and Cancer networks

Regarding the number of permutations used for the permutation test applied on the Pearson's correlation networks, we evaluated its effect on the number of remaining network edges. We repeatedly performed the permutation test varying the times of shuffling in a range from 5 to 100. For the LIHC tumor Normal and Cancer networks, Supplementary Tables 2,3 show that the remaining

number of edges after the permutation tests from 10 to 100 times shuffling is the same; thus, to speed up the process, we considered 10 times shuffling. Supplementary Tables 2,3 show also the used thresholds in the permutation tests with the different shuffling times. Particularly, the two low and high thresholds indicate the lower and higher limit values of the weights of the removed network edges: if an edge has a Pearson's correlation weight lower than threshold high and higher than threshold low, it is removed from the network.

Supplementary Table 2: Permutation tests on LIHC Normal network.

N^o **shuffling**	**Threshold low**	**Threshold high**	N^o **edges remaining**
5	-0.169	0.026	53,112,782
10	-0.158	-0.066	33,670,786
20	-0.134	-0.045	33,670,786
50	-0.134	-0.045	33,670,786
100	-0.134	-0.045	33,670,786

Supplementary Table 3: Permutation tests on LIHC Cancer network.

N^o **shuffling**	**Threshold low**	**Threshold high**	N^o **edges remaining**
5	-0.097	0.072	88,683,286
10	-0.035	0.037	74,027,936
20	-0.031	0.037	74,027,936
50	-0.031	0.037	74,027,936
100	-0.031	0.037	74,027,936

References

[1] Saint-Antoine, M. M. & Singh, A. Network inference in systems biology: recent developments, challenges, and applications. *Curr Opin Biotechnol* **63**, 89–98 (2020).

[2] Ptashne, M. The chemistry of regulation of genes and other things. *J Biol Chem* **289**, 5417–5435 (2014).

[3] Karlebach, G. & Shamir, R. Modelling and analysis of gene regulatory networks. *Nat Rev Mol Cell Biol* **9**, 770–780 (2008).

[4] Kreeger, P. K. & Lauffenburger, D. A. Cancer systems biology: a network modeling perspective. *Carcinogenesis* **31**, 2–8 (2010).

[5] Zhang, B. & Horvath, S. A general framework for weighted gene co-expression network analysis. *Stat Appl Genet Mol Biol* **4**, Article17 (2005).

[6] Marbach, D., Costello, J. C., Küffner, R. *et al.* Wisdom of crowds for robust gene network inference. *Nat Methods* **9**, 796–804 (2012).

[7] Care, M. A., Westhead, D. R. & Tooze, R. M. Parsimonious Gene Correlation Network Analysis (PGCNA): a tool to define modular gene co-expression for refined molecular stratification in cancer. *NPJ Syst Biol Appl* **5**, 1–17 (2019).

[8] Haury, A. C., Mordelet, F., Vera Licona, P. *et al.* TIGRESS: trustful inference of gene regulation using stability selection. *BMC Syst Biol* **6**, 145 (2012).

[9] Vân Anh Huynh Thu, A. I., Wehenkel, L. & Geurts, P. Inferring regulatory networks from expression data using tree-based methods. *PloS One* **5**, e12776 (2010).

[10] Singh, N. & Vidyasagar, M. bLARS: an algorithm to infer gene regulatory networks. *IEEE/ACM Trans Comput Biol Bioinform* **13**, 301–314 (2015).

[11] Friedman, N., Linial, M., Nachman, I. *et al.* Using Bayesian networks to analyze expression data. *J Comput Biol* **7**, 601–620 (2000).

[12] Yu, J., Smith, V. A., Wang, P. P. *et al.* Advances to Bayesian network inference for generating causal networks from observational biological data. *Bioinformatics* **20**, 3594–3603 (2004).

[13] Kraskov, A., Stögbauer, H. & Grassberger, P. Estimating mutual information. *Phys Rev E* **69**, 066138 (2004).

[14] Steuer, R., Kurths, J., Daub, C. O. *et al.* The mutual information: detecting and evaluating dependencies between variables. *Bioinformatics* **18**, S231–S240 (2002).

[15] Margolin, A. A., Nemenman, I., Basso, K. *et al.* ARACNE: an algorithm for the reconstruction of gene regulatory networks in a mammalian cellular context. *BMC Bioinformatics* **7**, S7 (2006).

[16] Butte, A. J. & Kohane, I. S. Mutual information relevance networks: functional genomic clustering using pairwise entropy measurements. In *Pac Symp Biocomput*, 418–429 (World Scientific, 2000).

[17] Faith, J. J., Hayete, B., Thaden, J. T. *et al.* Large-scale mapping and validation of Escherichia coli transcriptional regulation from a compendium of expression profiles. *PLoS Biol* **5**, e8 (2007).

[18] Meyer, P. E., Kontos, K., Lafitte, F. *et al.* Information-theoretic inference of large transcriptional regulatory networks. *EURASIP J Bioinform Syst Biol* **2007**, 1–9 (2007).
