# Supplementary material for: Computational analysis of fused co-expression networks for the identification of candidate cancer gene biomarkers
Source: NPJ Syst Biol Appl. 2021 Mar 12;7:17. doi: 10.1038/s41540-021-00175-9 (PMC7955132; doi:10.1038/s41540-021-00175-9)
Supplement: Supplementary file 2 — Reporting Summary [file 41540_2021_175_MOESM2_ESM.pdf]

nature research

Corresponding author(s): DBPR muniscript number: NPJSBA-00601R

Last updated by author(s): Dec 30, 2020

Reporting Summary

Nature Research wishes to improve the reproducibility of the work that we publish. This form provides structure for consistency and transparency in reporting. For further information on Nature Research policies, see our Editorial Policies and the Editorial Policy Checklist.

Statistics

For all statistical analyses, confirm that the following items are present in the figure legend, table legend, main text, or Methods section.

n/a	Confirmed	
☐	☒	The exact sample size (*n*) for each experimental group/condition, given as a discrete number and unit of measurement
☐	☒	A statement on whether measurements were taken from distinct samples or whether the same sample was measured repeatedly
☐	☒	The statistical test(s) used AND whether they are one- or two-sided<br>*Only common tests should be described solely by name; describe more complex techniques in the Methods section.*
☐	☒	A description of all covariates tested
☐	☒	A description of any assumptions or corrections, such as tests of normality and adjustment for multiple comparisons
☐	☒	A full description of the statistical parameters including central tendency (e.g. means) or other basic estimates (e.g. regression coefficient) AND variation (e.g. standard deviation) or associated estimates of uncertainty (e.g. confidence intervals)
☐	☒	For null hypothesis testing, the test statistic (e.g. *F*, *t*, *r*) with confidence intervals, effect sizes, degrees of freedom and *P* value noted<br>*Give P values as exact values whenever suitable.*
☒	☐	For Bayesian analysis, information on the choice of priors and Markov chain Monte Carlo settings
☒	☐	For hierarchical and complex designs, identification of the appropriate level for tests and full reporting of outcomes
☐	☒	Estimates of effect sizes (e.g. Cohen's *d*, Pearson's *r*), indicating how they were calculated

Our web collection on statistics for biologists contains articles on many of the points above.

Software and code

Policy information about availability of computer code

Data collection	Users can find the primary data and the complete pipeline for data extraction and preprocess in https://github.com/DEIB-GECO/GeneNetFusion. Data are collected either in text format or NumPy format (which is created through the NumPy python library).
Data analysis	Data analysis has been made using python language, all scripts and dependencies are available in in https://github.com/DEIB-GECO/GeneNetFusion. We also provide a Jupyter Notebook for an easy execution of all the steps of the developed computational pipeline.

For manuscripts utilizing custom algorithms or software that are central to the research but not yet described in published literature, software must be made available to editors and reviewers. We strongly encourage code deposition in a community repository (e.g. GitHub). See the Nature Research guidelines for submitting code & software for further information.

Data

Policy information about availability of data

All manuscripts must include a data availability statement. This statement should provide the following information, where applicable:

- Accession codes, unique identifiers, or web links for publicly available datasets
- A list of figures that have associated raw data
- A description of any restrictions on data availability

We used the Python programming language to implement the main steps of our computational method in separate scripts, respectively regarding the data preprocessing, matrix creation, and matrix fusion, as well as the feature selection and cancer classification evaluation. All developed software and the Jupyter Notebook are open source and publicly available at https://github.com/DEIB-GECO/GeneNetFusion, where for all cancer types considered we also provide the list of our identified candidate biomarkers extracted from each of the fused networks.

Field-specific reporting

Please select the one below that is the best fit for your research. If you are not sure, read the appropriate sections before making your selection.

☒ Life sciences ☐ Behavioural & social sciences ☐ Ecological, evolutionary & environmental sciences

For a reference copy of the document with all sections, see nature.com/documents/nr-reporting-summary-flat.pdf

Life sciences study design

All studies must disclose on these points even when the disclosure is negative.

Sample size	Our study is based on The Cancer Genome Atlas (TCGA) datasets, which are publicly available. More in detail, we extracted all samples (normal and cancer) for LIHC, KIRC and PRAD cancer types, respectively. We decided to evaluate our pipeline on these datasets because they have a considerable number of samples.
Data exclusions	We considered expression data associated to messenger RNAs, long non-conding RNAs and microRNAs. Thus, all other RNAs expression data were excluded.
Replication	We performed some tests to evaluate our pipeline's robustness, mainly checking our results' variability with differents number of considered samples. We also tested the reliability of our pre-processing step by varying the main parameters of the applied methods.
Randomization	Normal and cancer samples are reported as such by The Cancer Genome Atlas consortium. The clinical relevance of their study is recognized worldwide.
Blinding	Each clinical and expression sample data from the TCGA project is referred to a specific barcode, which ensures the privacy of the patient and uniquely identifies a set of results for a particular sample.

Reporting for specific materials, systems and methods

We require information from authors about some types of materials, experimental systems and methods used in many studies. Here, indicate whether each material, system or method listed is relevant to your study. If you are not sure if a list item applies to your research, read the appropriate section before selecting a response.

Materials & experimental systems

n/a	Involved in the study	
☒	☐	Antibodies
☒	☐	Eukaryotic cell lines
☒	☐	Palaeontology and archaeology
☒	☐	Animals and other organisms
☒	☐	Human research participants
☒	☐	Clinical data
☒	☐	Dual use research of concern

Methods

n/a	Involved in the study	
☒	☐	ChIP-seq
☒	☐	Flow cytometry
☒	☐	MRI-based neuroimaging
